# Association between Lifelines Diet Score (LLDS) and some novel anthropometric indices, including Body Roundness Index (BRI), A Body Shape Index (ABSI), Visceral Adiposity Index (VAI), and Body Adiposity Index (BAI), in Iranian women: a cross-sectional study

**DOI:** 10.1186/s12905-024-03013-2

**Published:** 2024-03-12

**Authors:** Matin Nazari, Khadijeh Mirzaie, Seyedali Keshavarz

**Affiliations:** 1 Department of Nutrition, Science and Research Branch, Islamic Azad University, Tehran, Iran; 2 Department of Community Nutrition, School of Nutritional Sciences and Dietetics, Tehran University of Medical Sciences (TUMS), Tehran, Iran

**Keywords:** Lifeline diet score, Anthropometry, BRI, ABSI, VAI, BAI

## Abstract

**Background:**

Anthropometry is a reliable method to assess obesity status, and previous studies have shown the association of several dietary quality scores with obesity using anthropometric indices. This study aimed to evaluate the association between LLDS and anthropometric measurements.

**Methods:**

A total of 217 women between the ages of 18 and 48 participated in the study. Anthropometric values, biochemical tests, and body composition were assessed for each participant using standard protocols and methods. The LLDS was determined based on 12 components using a reliable and valid food frequency questionnaire (FFQ) that contained 147 items.

**Results:**

We detected a marginally significant inverse association between the LLDS and VAI scores in the second tertile. Study participants in the second tertile of LLDS had lower odds of having high VAI than those in the reference tertile after adjusting for age, energy intake, physical activity, education, and economic status (*OR: -0.16; 95% CI: -0.8, 0.06; P = 0.06*). There was no statistically significant trend for the association between LLDS and all assessed anthropometric indices, including BRI, ABSI, VAI, and BAI, across tertiles of LLDS in the crude and all adjusted models (*P-trend > 0.05*).

**Conclusions:**

There was no significant association between LLDS and some novel anthropometric indices, including BRI, ABSI, VAI, and BAI. However, after adjusting for probable confounders, a marginally significant inverse association between LLDS and VAI was detected.

## Background

Obesity, the sixth health burden in the world, is a multifaceted phenomenon that reduces the quality of life and life expectancy and increases the risk of non-communicable diseases [1–3]. During the last century, our lifestyles and dietary habits have significantly changed, leading to the obesogenic environment we live in today. The growth of Western lifestyles characterizes this transition and causes an increase in overweight and obesity worldwide [2, 4].

Anthropometry is a reliable, effective, and affordable method for predicting the risk of cardiovascular disease, metabolic syndrome, and prevalence of overweight and obesity across populations and individuals [5]. Traditionally, despite the Body Mass Index’s (BMI) inability to discriminate between muscle and fat mass, it is the most commonly used index in epidemiological and clinical studies [6]. Additionally, due to its simplicity, BMI can lead to inaccurate assessment of the prevalence of overweight and obesity and underestimate the risk of developing obesity-related disease [7]. There is a paradoxical association between BMI and all-cause as well as disease-specific mortality in some chronic diseases like chronic kidney disease and cardiovascular diseases [8, 9]. Hence, studies have developed other anthropometric indices to more accurately assess obesity status, fat accumulation, and metabolic health to provide more predictive power. A Body Shape Index (ABSI) in 2012 and Body Roundness Index (BRI) in 2013 were proposed, combining traditional measurements such as height and waist circumference to more accurately predict the risk of pathologies that other anthropometric indices cannot indicate [10, 11]. Metabolic syndrome and hypertension have been shown to be associated with ABSI and BRI. Additionally, studies have suggested an association between ABSI and all-cause mortality and diabetes [11]. Based on this evidence, the importance of adipose tissue and its association with noncommunicable diseases cannot be neglected [12]. Hence, more specific and sensitive criteria, such as Visceral adiposity index (VAI), and Body Adiposity Index (BAI), are needed to accurately assess adipose tissue [13].

Based on recent nutritional studies, examining single nutrients and individual dietary components due to their synergistic or antagonistic effect in a mixed diet is not enough, and it is recommended to focus on dietary patterns in nutritional examinations. The relationship between different food patterns and their health benefits has been investigated. For instance, studies have suggested that the Mediterranean diet prevents chronic disease and early death [14]. The Lifelines Diet Score (LLDS) was recently developed based on Dutch dietary guidelines for 2015 to determine the relative quality of the diet. In the LLDS, foods are divided into positive and negative categories based on their health effects. Previous studies have demonstrated an association between higher LLDS and reducing the risk of some chronic diseases, such as breast cancer and unhealthy metabolic phenotypes [15, 16].

This study aimed to evaluate the association between LLDS and anthropometric measurements. We hypothesized that consuming a diet with higher LLDS because of following a healthier food pattern can effectively prevent weight gain and control obesity.

## Methods

### Study population

A group of 217 women between 18 and 48 years old participated in the current study. The target population was randomly selected from adult obese women referred to the Tehran University of Medical Sciences (TUMS) health centers. All participants signed a written consent form. The study protocol was approved by the ethics committee of TUMS and Islamic Azad University with the following identification: IR.IAU.SRB.REC.1400.231. A proficient nutritionist acquired all anthropometric evaluations and measurements. The participants were needed to possess a body mass index (BMI) of 25 kg/m2 or greater to be eligible for inclusion in the study. Individuals with acute or chronic diseases, such as cardiovascular disease, diabetes, cancer, thyroid or kidney disease, as well as those who were pregnant, breastfeeding, going through menopause, consuming alcohol or smoking, adhering to a special diet, and regularly taking medications or supplements (triglyceride-lowering drugs or supplements), were excluded as part of the study’s exclusion criteria. The study did not include individuals with energy intake below 800 kcal/d or above 4200 kcal/d.

### Body composition and anthropometric measurement assessment

The study assessed body composition using the InBody 770 scanner and measured weight using calibrated digital scales and height using a wall-mounted stadiometer [17]. Additionally, Waist Circumference (WC) and hip circumference (HC) were calculated precisely. BMI was also calculated as weight divided by height squared.

ABSI was calculated using the following formula [18]:

$$^{ABSI{\mkern 1mu} = {\mkern 1mu} WC/\left[ {{{\left( {BMI} \right)}^ \wedge }{\mkern 1mu} \left( {2/3} \right){\mkern 1mu} \times {\mkern 1mu} {{\left( {height} \right)}^ \wedge }{\mkern 1mu} \left( {1/2} \right)} \right]}$$

BRI was calculated using the following formula [19]:

$$\begin{array}{*{20}{l}}{{\rm{BRI}}\,{\rm{ = }}\,{\rm{365}}.{\rm{2}} - {\rm{365}}.{\rm{5}}}\\{ \times \sqrt {(1 - (((wc/2\pi )2)/{{[(0.5 \times height)]}^ \wedge }2))} }\end{array}$$

VAI was calculated using the following formula [20]:

$$\begin{array}{l}{\rm{Men:}}\,{\rm{VAI}}\,{\rm{ = }}\,\left[ {{\rm{WC/39}}{\rm{.68}}\,{\rm{ + }}\,{\rm{(1}}{\rm{.88}}\,{\rm{ \times }}\,{\rm{body}}\,{\rm{mass}}\,{\rm{index}}\,} \right[{\rm{BMI}}\left] {\rm{)}} \right]\,\\{\rm{ \times }}\,\left[ {{\rm{triglycerides}}\,\left( {{\rm{TG}}} \right){\rm{/1}}{\rm{.03}}} \right]\,{\rm{ \times }}\,\left( {{\rm{1}}{\rm{.31/HDL}}} \right){\rm{;}}\end{array}$$

$$\begin{array}{l}{\rm{women:}}\,{\rm{VAI}}\,{\rm{ = }}\,\left[ {{\rm{WC/36}}{\rm{.58}}\,{\rm{ + }}\,\left( {{\rm{1}}{\rm{.89}}\,{\rm{ \times }}\,{\rm{BMI}}} \right)} \right]\\{\rm{ \times }}\,\left( {{\rm{TG/0}}{\rm{.81}}} \right)\,{\rm{ \times }}\,\left( {{\rm{1}}{\rm{.52/HDL}}} \right){\rm{.}}\end{array}$$

Both TG and HDL levels are expressed in mmol/L.

BAI was calculated using the following formula [21]:

$$\begin{array}{l}{\rm{BAI}}\,\left[ {\% \,{\rm{body}}\,{\rm{fat}}} \right]\,{\rm{ = }}\,({\rm{Hipcircumference}}\left[ {{\rm{cm}}} \right]{\rm{/}}\\\left( {{\rm{Height}}{\mkern 1mu} \left[ {\rm{m}} \right]} \right){\rm{1}}.{\rm{5}}) - {\rm{18}}\end{array}$$

### Assessment of dietary intake

We assessed the dietary intake of all participants using a semiquantitative food frequency questionnaire (FFQ) that included a list of 147 food items consumed over the past year. The amount and frequency of each food item consumed daily, weekly, or monthly was reported by all participants. The frequency of consumption for each food item was recorded and subsequently converted to grams per day The dietary nutrient intake was evaluated using N4 (First Data Bank, San Bruno, CA) software, which had its database adapted for Iranian foods. This questionnaire was found to have high validity and reliability, ensuring the accuracy of the results [22].

### Lifelines diet score

The LLDS estimates dietary quality based on a Vinke et al. method used to rank people regarding relative food quality [23]. Overall, according to the 2015 Dutch diet guidelines, which are entirely based on scientific evidence, LLDS includes the consumption of nine food groups of vegetables, fruit, whole grain products, legumes and nuts, fish, oils and soft margarine, unsweetened dairy, coffee, and tea that have been proven to have positive effects on health, and three food groups including red and processed meat, butter and hard margarine, and sugar-sweetened beverages that have a negative impact on health. To calculate the LLDS score, we performed energy adjustment by computing food consumption for each individual in grams per 1000 kcal. Food intake of the participants was divided into 1 to 5 quintiles. The positive group’s maximum intake was rated 5 points, while the minimum intake of positive food groups was awarded 1 point. Similarly, for the negative food group, the highest intake was rated 1 point, and the lowest intake was rated 5 points. The study’s results were summarized using the LLDS score, which ranges from 12 to 60, and reflects the scores of all twelve components [23, 24]. .

### Assessment of other variables

We used a validated International Physical Activity Questionnaire (IPAQ) to determine how physically active the participants were. According to the IPAQ scoring protocol, individuals were divided into the following groups in terms of physical activity: [1] low active (< 600 MET (Metabolic Equivalent of Task) -h/week); [2] moderate active (≥ 600 MET-h/week); and [3] high active (≥ 3000 MET-h/week). We also used a standard sociodemographic questionnaire to collect data on age, education level, marital status, job, supplementation, and economic status.

### Statistical analysis

The calculation of the sample size was based on a study conducted among adults from Iran. In this study, the standard deviation (SD) of the dietary phytochemical index, which can be viewed as comparable to the LLDS, was reported to be 7.5 [25]. Furthermore, the correlation between a healthy dietary pattern and BMI in Iranian women was found to be -0.73 [26]. Given an alpha error of 5% and a power of 80%, an estimated sample size of approximately 122 participants would be required for our study.

The Kolmogorov–Smirnov test evaluated the distribution of variables, which was normal. Data on continuous characteristics were reported as the mean ± standard deviation (SD), and data on categorical characteristics were expressed as percentages and numbers. The chi-square test was used to evaluate significant differences in categorical variables among tertiles of the LLDS score, and one-way analysis of variance (ANOVA) was used to assess significant mean differences in continuous variables across tertiles of the LLDS cutoff points (T1: ≤34, T2: 35–38, T3 ≥ 39). Bonferroni’s post hoc multiple comparison analysis showed a significant mean difference between the groups. Analysis of covariance (ANCOVA) was used to identify dietary intakes and general characteristics mean differences between tertiles of the LLDS after being adjusted by energy intake for the dietary intakes and further with age, physical activity, and BMI for general characteristics. We used linear regression to assess the association between the LLDS score and the BRI, VAI, ABSI, and BAI in crude and multivariable models. Age, energy intake, and physical activity were controlled for in the first model. Further adjustment was made for education and economic status in the second model. The first tertile of the LLDS score was considered the reference category. In the present study, *P* values < 0.05 and *P* values = 0.05, 0.06, and 0.07 were considered significant levels and marginally significant, respectively. All statistical analyses via the statistical package for social sciences (version 24; SPSS Inc., Chicago, IL, USA) were performed.

## Results

### Characteristics of the study population

Table 1 demonstrates baseline characteristics of the study subjects across tertiles of the LLDS. There was no significant mean difference in terms of physical activity, education, economic status, serum levels of insulin and HDL-C, or anthropometric indices, including BMI, Waist-Hip Ratio (WHR), Fat Free Mass Index (FFMI), BRI, ABSI, AVI, and BAI, across tertiles of LLDS (*P > 0.05*). However, we found a significant difference in age and Fat Free Mass (FFM) among tertiles of LLDS in the crude model (*P = 0.06* and *P* = 0.04, respectively). Additionally, after adjusting for probable confounders, including age, energy intake, physical activity, and BMI, there was a significant difference in age across tertiles of LLDS (*P = 0.03*). Based on the post hoc analysis, the mean age of the participants in the third tertile of the LLDS was greater than that in the first tertile of the LLDS.

**Table 1:** General characteristics of study population among tertiles of the LLDS score

Variables	Tertiles of the LLDS score				*P* value	*P* value *
	T_1_ (*n* = 76) ≤ 34	T_2_ (*n* = 80) 35–38		T_3_ (*n* = 61) ≥ 39		
Quantitative variable	Mean ± SD					
**Demographic characteristic**						
Age (Y)	34.66 ± 8.66 ^a^		35.89 ± 8.36	38.08 ± 7.98 ^a^	**0.06**	**0.03**
PA (MET-min/week)	1046.03 ± 1953.25		1058.70 ± 1287.57	1389.63 ± 2207.04	0.50	0.45
**Anthropometry and Body Composition**						
Weight (kg)	78.81 ± 11.39		78.44 ± 9.10	81.03 ± 10.72	0.30	0.10
Height (cm)	162.27 ± 5.57		160.32 ± 5.57	161.29 ± 5.47	0.09	0.75
WC (cm)	95.22 ± 10.33		93.32 ± 15.81	96.22 ± 96.22	0.64	0.64
HC (cm)	112.93 ± 9.17		112.96 ± 6.90	114.29 ± 8.14	0.62	0.50
BMI (kg/)	29.91 ± 3.71		30.65 ± 3.47	30.98 ± 3.44	0.18	0.23
WHR	0.93 ± 0.05		0.92 ± 0.04	0.93 ± 0.05	0.81	0.41
BF (%)	40.75 ± 4.41		41.58 ± 5.08	40.94 ± 4.99	0.70	0.99
BFM (kg)	32.36 ± 7.39		32.94 ± 6.57	33.35 ± 7.37	0.16	0.62
FFM (kg)	46.36 ± 5.29		45.81 ± 5.35	47.48 ± 5.62	**0.04**	0.44
VFL	15.03 ± 3.22		15.43 ± 3.23	15.37 ± 3.33	0.69	0.43
FMI (kg)	12.30 ± 2.70		12.87 ± 2.75	13.01 ± 3.00	0.29	0.71
FFMI (kg)	17.60 ± 1.46		17.75 ± 1.50	18.19 ± 1.34	0.24	0.36
**Categorical variables** ^*^						
**Education**					0.41	0.40
Under diploma	9 (37.5)	7 (29.2)		8 (33.3)		
Diploma	19 (29.7)	22 (34.4)		23 (35.9)		
Above diploma	46 (36.2)	51 (40.2)		30 (23.6)		
**Marital status**					0.68	0.31
Single	17 (36.2)	19 (40.4)		11 (23.4)		
Married	57 (33.9)	61 (36.3)		50 (29.8)		
**Job**					0.54	0.13
Non-employed	44 (32.4)	51 (37.5)		41 (30.1)		
Employed	29 (38.7)	28 (37.3)		18 (24.0)		
**Economic status**					0.22	0.19
Poor	14 (25.5)	23 (41.8)		18 (32.7)		
Moderate	32 (34.8)	39 (42.4)		21 (22.8)		
Good	24 (42.9)	16 (28.6)		16 (28.6)		

BF%; body fat percentage; BFM: body fat mass; BMI: body mass index; FFM: fat free mass; FFMI; fat free mass index; FMI; fat mass index; HC: hip circumference; LLDS: Lifelines Diet Score; PA: physical activity; SD: Standard Deviation; T: tertile; VFL: visceral fat level; WC: waist circumference; WHR: waist-hip ratio

*P* value: ANOVA test was used

*P* value*: ANCOVA was performed to adjusted potential confounding factors (age, energy intake, Physical activity, BMI), BMI consider as collinear variable for anthropometrics and body composition variables

Chi-square was used for categorical variables

*P*-values < 0.05 were considered as significant

Values are represented as means ± SD

categorical variables: N (%)

^a^ The significant difference was seen between T_1_ and T_3_

### Dietary intake of study participants

Table 2 shows the dietary intakes of study subjects, categorized based on the tertiles of LLDS. There was no significant mean difference in terms of whole grain and tea consumption across tertiles of LLDS (*P > 0.05*). However, we found a significant difference in the consumption of vegetables, fruits, legumes and nuts, fish, coffee, unsweetened dairy, oils, soft margarine, red and processed meat, sugar-sweetened beverages, and butter and hard margarine among tertiles of LLDS in the crude model and after adjusting for energy intake (*P < 0.04*). Study participants in the third tertile of LLDS had significantly higher intakes of vegetables, legumes, nuts, fish, unsweetened dairy (*P* < 0.001), fruits (*P* = 0.001), oils, soft margarine (*P* = 0.009), and coffee (*P* = 0.004). However, the data show significantly lower intakes of red and processed meat (*P* = 0.01), butter and hard margarine (*P* < 0.001), and sugar-sweetened beverages (*P* = 0.005).

**Table 2:** Dietary intake of study participants across tertiles of LLDS

Variables	Tertiles of LLDS			*P* value	*P* value ^*^
	T1	T2	T3		
**Food groups**	Mean ± SD	Mean ± SD	Mean ± SD		
**Positive food groups**					
Vegetables (g/d)	357.31 ± 256.94	424.34 ± 256.34	572.62 ± 254.26	**< 0.001**	**< 0.001**
Fruits (g/d)	504.46 ± 332.35	567.62 ± 365.52	668.98 ± 425.40	**0.03**	**0.001**
Legume and nuts (g/d)	51.06 ± 30.16	61.53 ± 46.22	87.12 ± 53.87	**< 0.001**	**< 0.001**
Whole grain	450.27 ± 202.69	388.78 ± 155.21	467.14 ± 306.79	0.08	0.29
Fish	9.37 ± 11.85	9.71 ± 7.77	17.30 ± 14.48	**< 0.001**	**< 0.001**
Coffee	10.21 ± 26.19	22.44 ± 48.35	39.35 ± 71.45	**0.004**	**0.004**
Tea	764.44 ± 595.17	620.52 ± 476.88	805.71 ± 548.39	0.09	0.20
Unsweetened dairy	270.81 ± 232.52	333.90 ± 215.08	442.59 ± 264.84	**< 0.001**	**< 0.001**
Oils and soft margarines	12.54 ± 13.44	17.90 ± 15.91	20.22 ± 17.47	**0.01**	**0.009**
**Negative food groups**					
Red and processed meat	37.59 ± 24.84	28.25 ± 26.01	25.62 ± 19.55	**0.008**	**0.01**
Sugar-sweetened beverages	41.47 ± 48.41	29.10 ± 78.30	9.68 ± 15.36	**0.005**	**0.005**
Butter and hard margarines	25.10 ± 26.43	7.99 ± 12.66	3.70 ± 7.81	**< 0.001**	**< 0.001**
**Macronutrients**					
Energy (kcal)	2725.86 ± 813.05	2497.91 ± 762.85	2674.12 ± 663.23	0.14	-
Carbohydrates (g/d)	374.39 ± 125.36	359.81 ± 119.95	394.51 ± 122.16	0.25	**0.001**
Total Fat (g/d)	105.34 ± 36.44	88.64 ± 32.95	88.50 ± 22.46	**0.001**	**< 0.001**
Protein (g/d)	87.15 ± 31.60	84.78 ± 25.82	98.08 ± 23.73	**0.01**	**< 0.001**

SD: Standard Deviation; T: tertile

*P* value: ANOVA test was used

*P* value*: ANCOVA was performed to adjusted potential confounding factors (energy intake)

Values are represented as means ± SD

*P*-values < 0.05 were considered as significant

### Association between LLDS score and some novel anthropometric indices

Table 3 shows the association between LLDS and anthropometric indices, including BRI, ABSI, VAI, and BAI. There was no significant association between LLDS and any of the assessed anthropometric indices in the crude model. Additionally, in Model 1 and Model 2, after adjusting for probable confounders, including age, energy intake, physical activity, education, and economic status, no significant association between LLDS and the mentioned anthropometric indices except VAI was found. Regarding VAI, we detected a marginally significant inverse association between the second tertile of LLDS and VAI in Model 2. Study participants in the second tertile of LLDS had lower odds of having high VAI than those in the reference tertile after adjusting for age, energy intake, physical activity, education, and economic status (*OR: -0.16; 95% CI: -0.8, 0.06; P = 0.06*). Additionally, there was no statistically significant trend for the association between LLDS and all assessed anthropometric indices, including BRI, ABSI, VAI, and BAI, across tertiles of LLDS in the crude and all adjusted models (*P-trend > 0.05*).

**Table 3:** The association between LLDS and some novel anthropometric indices

Anthropometric indices	Tertiles of LLDS						*P* trend^*^
	T1 (*n* = 76)		T2 (*n* = 80)		T3 (*n* = 61)		
	β (95% CI)	*P* value^*^	β (95% CI)	*P* value^*^	β (95% CI)	*P* value^*^	
**BRI**							
Crude	0	0	-0.093 (-0.47-0.28)	0.62	0.001 (-0.37-0.37)	0.99	0.984
Model 1	0	0	0.001 (-0.43-0.43)	1.0	0.133 (-0.29-0.56)	0.54	0.545
Model 2	0	0	0.07 (-0.37-0.51)	0.75	-0.201 (-0.24-0.64)	0.37	0.051
**ABSI**							
Crude	0	0	-2.6 (-0.001-0.001)	0.95	0.001 (-0.001-0.001)	0.72	0.72
Model 1	0	0	0.001 (-0.001-0.001)	0.78	4.8 (-0.001-0.001)	0.91	0.91
Model 2	0	0	6.55 (-0.001-0.001)	0.89	-9.9 (-0.001-0.001)	0.84	0.84
**VAI**							
Crude	0	0	-0.08 (-0.67-0.5)	0.77	0.24 (-0.31-0.81)	0.39	0.36
Model 1	0	0	-0.19 (-0.88-0.5)	0.59	0.28 (-0.39-0.96)	0.41	0.38
Model 2	0	0	-0.16 (-0.8-0.06)	**0.06**	0.16 (-0.46-0.78)	0.6	0.59
**BAI**							
Crude	0	0	0.69 (-2.4-3.8)	0.66	-0.18 (-3.1-2.7)	0.89	0.8
Model 1	0	0	0.008 (-3.7-3.7)	0.99	-0.005 (-3.5-3.5)	0.99	0.99
Model 2	0	0	0.36 (-3.6-4.3)	0.85	0.001 (-3.7-3.7)	1.0	0.97

^*^ based on the linear regression test

Model 1: Adjusted for age, energy intake, and physical activity

Model 2: Model 1 further adjusted with education and economic status

## Discussion

As we know, despite the high prevalence of obesity in women [27], no previous study has evaluated the association between LLDS and anthropometric indices in Iranian women. For the first time, this investigation assessed the association between LLDS and some novel anthropometric indices, including BRI, ABSI, VAI, and BAI, in Iranian women. Based on the main obtained findings, there was no significant association between LLDS and anthropometric indices, including BRI, ABSI, and BAI. However, a marginally significant inverse association between the second tertile of LLDS and VAI was detected after adjusting for probable confounders. Additionally, no statistically significant trend for the association between LLDS and all assessed anthropometric indices across tertiles of LLDS was found.

Consistent with the findings of the present investigation, a recent cross-sectional study found no significant association between dietary patterns, mainly including red meats, eggs, and dairy, and the risk of overweight/obesity or underweight/wasting among Iranian children aged six years. Furthermore, there was no remarkable association between dietary patterns, mainly soy and legumes, and the risk of overweight/obesity or underweight/wasting [28]. Based on the conclusion of an investigation performed by Giontella et al. [29], there was no significant correlation between anthropometric indices and the consumption of vegetables, fruits, eggs, meat, dairy products, sweets, legumes, fish, and nuts. Additionally, a recent cross-sectional study on Iranian adults exhibited no significant association between major dietary patterns at dinner, including Prudent and Western patterns, and general or central obesity [30]. In addition, an investigation by Khadem et al. [16] on the same population as ours proposed no significant association between LLDS and obesity phenotypes. In contrast, in an 8-year prospective cohort study among women, there was a significant direct association between Western dietary patterns, reflected in diets high in red and processed meats and sweets, and worsened anthropometric indices [31]. Additionally, Slattery et al. [32] demonstrated a significant positive association between Western dietary patterns and obesity. Moreover, based on a cross-sectional study by Kim et al. [33], there was a significant direct association between diets containing red and processed meats and worsened anthropometric indices in Korean adults. In addition, a healthy dietary pattern rich in fruits, vegetables, and whole grains, such as ours, was proposed to reduce weight gain and improve body composition compared to the regular US diet in the Women’s Health Initiative study [34]. The discrepancies between our findings and the results mentioned earlier may result from different study designs, dissimilar study sizes, various study populations from different ethnic groups, diverse statistical methods for analyzing the results, and considering other possible confounders to adjust for statistical analysis.

According to the Dutch dietary guidelines, LLDS represents the quality of the diet, and in the case of increasing LLDS, the diet will be healthier [35]. The possible mechanisms responsible for the positive association between healthy dietary patterns, including LLDS and better anthropometric indices, were investigated in several prior studies [31, 36, 37]. A healthy diet rich in vegetables, fruits, nuts, whole grains, and unsaturated fatty acids, including Polyunsaturated Fatty Acids (PUFA) and Monounsaturated Fatty Acids (MUFA), was reported to reduce the risk of mortality [38], as well as physical frailty [39], and improve weight and anthropometric indices by regulating energy hemostasis, upregulating PPAR-α expression, inducing fatty acid β-oxidation, inhibiting the expression of lipogenesis genes, and increasing insulin sensitivity [36, 37, 40, 41]. However, an unhealthy diet rich in sweetened beverages, saturated fatty acids existing in butter and hard margarines, and red and processed meats was suggested to worsen anthropometric indices by increasing circulating leptin concentrations, decreasing the lipid handling capacity of adipocytes through suppression of fat oxidation, and exacerbating insulin resistance because of its higher glycemic index components and lower fiber content [36, 42–47].

### Study strengths and limitations

In the present investigation, despite several strong points, including using a validated FFQ for the Iranian population, examining this association for the first time in Iranian people, running robust statistical analysis, and acceptable study size, some limitations should also be pointed out to interpret our conclusion. First, the mentioned association was only evaluated in one gender and city. Therefore, the results could not be generalizable to both genders and the entire Iranian population. Second, due to the cross-sectional nature of the current research, it could not exhibit a causal effect. Third, although some probable confounding factors were adjusted, numerous other confounders may remain that could affect the results. Finally, measurement accuracy errors caused by applying FFQ for dietary assessment might influence the conclusion.

## Conclusions

There was no significant association between LLDS and some novel anthropometric indices, including BRI, ABSI, and BAI in our female population. However, a marginally significant inverse association between the second tertile of LLDS and VAI was detected after adjusting for probable confounders. Further studies with larger sample sizes and better study designs are recommended to investigate the association between LLDS and novel anthropometric indices.

## Data Availability

The data that support the findings of this study are available from Khadijeh Mirzaei, but restrictions apply to the availability of these data, which were used under license for the current study and so are not publicly available. Data are, however, available from the authors upon reasonable request and with permission of Khadijeh Mirzaei.

## Abbreviations

ABSI: A Body Shape Index
BAI: Body Adiposity Index
BF%: Body fat percentage
BFM: Body fat mass
BMI: Body mass index
BMI: Body mass index
BRI: Boby roundness index
CIs: Confidence intervals
FFM: Fat free mass
FFMI: Fat free mass index
FMI: Fat mass index
HC: Hip circumference
IPAQ: International physical activity questionnaire
LLDS: Lifelines Diet Score
PA: Physical activity
SDs: Standard deviations
T: Tertile
VAI: Visceral Adiposity Index
VFL: Visceral fat level
WC: Waist circumference
WHR: Waist-hip ratio
